# High concentrations of polyunsaturated n–3 fatty acids in serum are inversely associated with risk of future incident venous thromboembolism – the HUNT cohort study

**DOI:** 10.1016/j.ajcnut.2025.08.008

**Published:** 2025-08-18

**Authors:** Magdalena Johansson, Sigrid K Brækkan, Guro F Giskeødegård, Kaitlin H Wade, Kristian D Hindberg, Nicholas Timpson, George Davey Smith, Kristian Hveem, Bjørn Olav Åsvold, Ben M Brumpton, John-Bjarne Hansen

**Affiliations:** 1 Skellefteå Research Unit, Department of Public Health and Clinical Medicine, Umeå University, Umeå, Sweden; 2 Thrombosis Research Group (TREC), Department of Clinical Medicine, UiT – The Arctic University of Norway, Tromsø, Norway; 3 Thrombosis Research Center (TREC), Division of Internal Medicine, University Hospital of North Norway, Tromsø, Norway; 4 Department of Public Health and Nursing, Norwegian University of Science and Technology (NTNU), Trondheim, Norway; 5 Department of Surgery, St. Olav’s University Hospital, Trondheim, Norway; 6 MRC Integrative Epidemiology Unit (IEU), Bristol Medical School, University of Bristol, Bristol, UK; 7 HUNT Center for Molecular and Clinical Epidemiology, Norwegian University of Science and Technology (NTNU), Trondheim, Norway; 8 Levanger Hospital, Nord-Trøndelag Hospital Trust, Levanger, Norway; 9 HUNT Research Center, Department of Public Health and Nursing, Norwegian University of Science and Technology (NTNU), Levanger, Norway; 10 Department of Endocrinology, Clinic of Medicine, St. Olav’s Hospital, Trondheim University Hospital, Trondheim, Norway; 11 Clinic of Medicine, St. Olav’s Hospital, Trondheim University Hospital, Trondheim, Norway

**Keywords:** venous thromboembolism, venous thrombosis, deep vein thrombosis, pulmonary embolism, omega-3 fatty acids, lipids, risk factor, prospective studies, cohort studies

## Abstract

**Background:**

Previous studies on the association between n–3 (ω-3) polyunsaturated fatty acids (n–3 PUFAs) and risk of venous thromboembolism (VTE), mainly derived from self-reported dietary intake of n–3 PUFAs, have shown mixed results.

**Objectives:**

The aim of this cohort study was to investigate the association between measured serum n–3 PUFA concentrations and risk of first-ever VTE.

**Methods:**

The present cohort (*N* = 17,087) was derived from the third survey of the Trøndelag Health Study and consisted of individuals aged ≥20 y without previous VTE. Serum n–3 PUFA concentrations were measured in blood drawn at inclusion (2006–2008) using a nuclear magnetic resonance metabolomic platform (Nightingale), and participants were followed until an objectively verified first-ever VTE event, death, migration, or 31 December, 2019. Cox regression was used to estimate hazard ratios (HRs) with 95% confidence intervals (CIs).

**Results:**

During a median follow-up of 12 y, 340 experienced an incident VTE event. Participants with n–3 PUFA concentrations in the highest tertile had 28% lower relative risk of VTE (HR: 0.72; 95% CI: 0.54, 0.96) than those with n–3 PUFAs in the lowest tertile in age-adjusted analysis. The inverse association was most pronounced for provoked VTE (HR: 0.62; 95% CI: 0.44, 0.87) and deep vein thrombosis (HR: 0.61; 95% CI: 0.40, 0.93). HRs remained virtually unaffected after further adjustment for sex, body mass index, and history of cardiovascular disease or cancer at baseline.

**Conclusions:**

Our finding of an inverse association between serum n–3 PUFA concentrations and VTE suggest that a diet enriched with n–3 PUFAs might protect against future VTE.

## Introduction

Venous thromboembolism (VTE), including deep vein thrombosis (DVT) and pulmonary embolism (PE), affects almost 10 million individuals worldwide annually [1]. The incidence rate (IR) of VTE has increased in recent decades [[2], [3], [4]], and VTE is a major health care challenge due to long-term complications, hospitalizations, comorbidities, and a high mortality rate [1,[5], [6], [7], [8]]. Therefore, it is imperative to identify lifestyle factors that protect against VTE to promote lifestyle changes that can potentially reduce the burden of VTE in society.

Marine n–3 PUFAs, e.g., EPA and DHA, are essential fatty acids acquired by seafood consumption [9]. n–3 PUFAs are associated with lowered inflammation [10], reduced thrombin generation [11], inhibition of platelet function [12,13], and less endothelial dysfunction [14], all of which might be implicated in VTE pathogenesis. Various n–3 PUFAs can have different biological effects [15] and are linked to biosynthesis pathways [16]. In humans, EPA does not seem to be converted to DHA, whereas DHA is retroconverted to EPA [15,17]. Serum n–3 PUFA concentrations, reflecting concentrations of EPA and DHA and other n–3 PUFAs, could be a useful measure when studying the relationship between dietary n–3 PUFA intake and health outcomes.

Studies on the association between fish consumption or estimated n–3 PUFA intake and VTE risk have shown mixed results [[18], [19], [20], [21], [22], [23], [24]]. Two cohort studies showed an association between higher self-reported fish consumption and lower risk of first-ever VTE [18,19], whereas in another cohort, a lower risk of idiopathic VTE observed in participants with higher intake of fatty fish was not statistically significant [20]. The mixed results may be due to misclassification of the exposure variable (e.g., fish intake and estimated n–3 PUFA intake), differences in study designs and populations, confounders, and VTE ascertainment. As the n–3 PUFA content varies substantially between different types of fish, farmed and wild caught fish, and between n–3 PUFA supplements, studies of health effects of n–3 PUFA intake entail a risk of nondifferential exposure misclassifications, which can lead to incorrect estimations of true associations [[25], [26], [27]]. Accurate measurements of serum n–3 PUFA, reflecting the dietary intake of n–3 PUFAs, would limit nondifferential misclassification. Two small case-control studies reported lower serum EPA concentrations in individuals with VTE [28,29], and a study derived from the UK Biobank that included prevalent and incident VTE cases showed that increased DHA and total n–3 PUFA concentrations were associated with lower odds of VTE [30]. To our knowledge, no previous study has investigated the association between serum total n–3 PUFAs and risk of future VTE. Therefore, we aimed to investigate *1*) to what extent estimated n–3 PUFA intake agreed with serum n–3 PUFA concentration and *2*) whether serum concentrations of n–3 PUFAs and DHA were associated with risk of future VTE.

## Methods

### Study population

The study is a prospective cohort study derived from the Trøndelag Health (HUNT) study. The HUNT study is a Norwegian follow-up study with repeated health surveys of inhabitants of the Nord-Trøndelag region, Norway [31,32]. The third HUNT survey (HUNT3) was conducted from August 2006 to June 2008. All inhabitants of Nord-Trøndelag aged ≥20 y were invited to participate in HUNT3, and 50,800 inhabitants (54.5% of those who were invited) participated in the survey. The present metabolomics study included an unselected sample of HUNT3 participants enrolled between August 2007 and June 2008 (*N* = 17,215). Those with previous VTE (*n* = 114), those who had officially moved from Nord-Trøndelag before inclusion (*n* = 6), and those with missing values in metabolomics data (*n* = 8) were excluded, rendering 17,087 participants in the final study sample. The participants were followed from the date of HUNT3 participation up to 31 December, 2019, and all first-ever VTE events were validated and recorded. All participants provided written informed consent for participation in HUNT3, and the study was approved by the Regional Committee for Medical and Health Research Ethics.

### HUNT3 baseline survey data and blood sampling

At the HUNT3 survey, baseline information was collected by physical examination, blood sampling, and questionnaires. Height and weight were measured, and BMI was calculated as body weight in kilograms divided by the square of the body height in meters. Information on history of cancer and cardiovascular disease, diabetes, smoking, physical activity, and intake of fruits, berries, and vegetables was obtained by questionnaires. Nonfasting venous blood samples were drawn into sample serum separation tubes (SSTs), left for 30 to 90 min at room temperature before centrifugation, and transported at 4°C to the HUNT Biobank. The next day, the SST samples were fractionated and dispersed into 2D Matrix tubes (Matrix 2D, Thermo Fisher Scientific) and subsequently stored at −80°C in the biobank [33].

### Dietary marine n–3 PUFA intake

Information about the intake of fish and fish oil supplements was collected by a questionnaire at the HUNT3 survey. The study participants answered the question, “How often do you normally eat these foods?” and were asked to score their intake of high-fat fish (salmon, trout, herring, mackerel, haddock) on bread or for dinner using the responses “0–3 times a month,” “1–3 times a week,” “4–6 times a week,” “once a day,” or “twice or more a day.” They also answered the question, “Do you take the following dietary supplements?” and were asked to separately score their intake of cod liver oil and omega-3 capsules using the responses “Yes, daily”, “Occasionally,” and “No.” Participants who did not answer the question on dietary supplements were regarded as not consuming such products. To estimate the content of marine n–3 PUFAs in each portion of fatty fish or from n–3 PUFA supplements, the estimated content of n–3 PUFAs of fatty fish or n–3 PUFA supplements was multiplied by a standard serving size according to recommendations from the Norwegian Directorate of Health, as previously described [34]. Then, the total weekly intake of marine n–3 PUFAs was calculated by multiplying the amount of marine n–3 PUFAs in each portion of fatty fish or n–3 PUFA supplement with the number of times per week that the food item or supplement in question was consumed. Frequencies reported as ranges in the questionnaire were recorded to the mean value of the range. The highest frequency option (twice or more a day) was recoded to twice a day. The weekly intakes of n–3 PUFAs from fatty fish, n–3 PUFA supplements, and fish oil were summed to obtain total marine n–3 PUFA intake.

### Quantification of serum n–3 PUFA concentrations

In 2019, samples were retrieved from the HUNT biobank, and concentrations of circulating DHA and n–3 PUFAs were measured in serum using a high-throughput nuclear magnetic resonance metabolomic platform (Nightingale Health UK Ltd) [35]. The Nightingale nuclear magnetic resonance spectroscopy method measures the total serum concentration of the entire fatty acid class, meaning the total concentration of all the molecules belonging to the class. Thus, in the present study, the term “n–3 PUFA concentration” encompasses all fatty acids with double bonds at the ω-3 position. Serum concentrations of DHA were estimated separately using the Nightingale platform.

The biobank samples were analyzed in 2 batches. The first batch (*n* = 6201 samples) was analyzed using a 600 MHz Bruker Avance spectrometer, and the second batch (*n* = 11,009 samples) was analyzed using one 500 MHz spectrometer and one 600 MHz spectrometer. Results from the analyses of the second batch were adjusted for instrument effects as follows. Samples from 96 individuals in the second batch were analyzed using both instruments. Results from the analyses of samples analyzed on the 500 MHz instrument were then multiplied by a unique correction factor for each variable, which resulted in equal median values for analyses from the 2 instruments. Finally, the results from the analyses of the second batch were adjusted toward those of the first batch by multiplying the results from the second batch with a correction factor for each variable. This resulted in equal means for the 2 batches. For n–3 PUFAs, the Nightingale metabolomics panel has previously been shown to have coefficients of variation of 5.0% (within spectrometer) and 6.6% (between spectrometers). For DHA, the respective coefficients of variation are 6.7% and 7.7% [36,37].

### VTE identification and validation

The catchment area of the HUNT3 participants is served by 3 hospitals: Levanger Hospital, Namsos Hospital, and St. Olav’s Hospital (Trondheim University Hospital). An extensive search in the discharge diagnosis registries of all 3 hospitals was performed for *International Classification of Diseases, Tenth Revision* codes related to VTE from 2008 to 2019 [38]. All potential VTE cases identified in the diagnosis registry search were manually validated by review of medical records. VTE events were considered verified if signs and symptoms of lower extremity DVT or PE were followed by objective confirmation of the diagnosis by radiological examinations (ultrasound, venography, or computed tomography) or autopsy, and treatment was initiated (unless contraindications for treatment were specified). If an individual simultaneously fulfilled the criteria for lower extremity DVT and PE, the event was classified as PE. Information about provoking factors for VTE was obtained from medical records using a standardized form. A VTE event was categorized as provoked if any of the following provoking factors was noted during 3 mo before the VTE event: surgery, trauma, acute myocardial infarction, stroke, a severe infection, immobilization, active cancer, or another factor specifically described as a provoking factor in the medical record. Immobilization was defined as bed rest for ≥3 d, need for a wheelchair, or plaster cast.

### Statistical methods

Baseline characteristics are reported as medians and interquartile ranges for continuous variables and as numbers and percentages for categorical variables. Spearman’s correlation coefficient was calculated to assess the correlation between dietary intake of marine n–3 PUFAs (grams per week), serum n–3 PUFA concentrations (millimoles per liter), and DHA concentrations (millimoles per liter). A linear regression analysis was used to assess the *R*^2^ value for the relationship between dietary intake of n–3 PUFAs and serum n–3 PUFA concentrations. κ statistics were used to assess the agreement between tertiles of self-reported weekly intake of n–3 PUFAs (grams per week) and tertiles of n–3 PUFA concentrations (millimoles per liter).

Person-time of follow-up for each individual was calculated from inclusion in HUNT3 until the first-ever VTE, migration from Nord-Trøndelag, death, or end of the study period (31 December, 2019), whichever occurred first. IRs of first-ever VTE were calculated for the entire study population as well as across tertiles of serum n–3 PUFA concentrations (millimoles per liter) and are expressed as the number of events per 1000 person-years.

Cox proportional hazards regression models with age as time scale were used to estimate hazard ratios (HRs) and 95% confidence intervals (CIs) for the associations between total serum n–3 PUFA concentrations (millimoles per liter), serum DHA concentrations (millimoles per liter), dietary marine n–3 PUFAs (grams per week) (estimated from self-reported fish and supplement intake), and risk of first-ever VTE. All determinants were analyzed both as continuous variables with HRs expressed per SD increase and as categorical variables in tertiles using the lowest tertile as reference. *P* values for linear trends over tertiles (entered as an ordinal variable) of serum n–3 PUFA concentrations (millimoles per liter), serum DHA concentrations (millimoles per liter), and dietary marine n–3 PUFAs (grams per week) were estimated. HRs were estimated for total first-ever VTE, for PE and DVT separately, and for provoked and unprovoked VTE separately. Two adjustment models were used. Model 1 was adjusted for age (as time scale). Model 2 was adjusted for age (as time scale), sex (male/female), BMI (in kilograms per meters squared as a continuous variable), and history of cardiovascular disease or cancer (presence or absence of history of cardiovascular disease or cancer). The proportional hazards assumption for the Cox proportional hazards model was tested using a global test based on the Schoenfeld residuals.

To assess potential nonlinearity between serum n–3 PUFA concentrations and risk of overall VTE, a generalized additive regression plot was generated to visualize the association by modeling total n–3 PUFA with a smoothing spline fit in a Cox model adjusted for the covariates of model 2 (using R version 4.4.2). The n–3 PUFA values were standardized to a mean value of 0 and a SD of 1 before entering the analyses.

Given the relatively long follow-up time in the parent cohort, the results based on baseline measurements of total n–3 PUFA concentrations could be influenced by regression dilution bias [39]. To address this, we performed analyses that restricted the maximum follow-up time from blood sampling to the VTE events, while keeping all cohort members in the analyses. The Cox regression analyses on time restrictions were set to require ≥10 VTE events, and HRs adjusted for the covariates included in model 2 were generated at every time point a new VTE event occurred and plotted as a function of this maximum time.

In a sensitivity analysis, participants with VTE provoked by active cancer were excluded from the analysis.

Individuals with missing data on a variable were excluded from analyses including that variable as a covariate (*n* = 6 for history of cardiovascular disease, *n* = 10 for history of cancer, *n* = 59 for BMI, and *n* = 413 for self-reported intake of marine n–3 PUFAs or number of weekly occasions of fatty fish consumption).

Statistical analyses were performed with STATA version 14.2 (Stata Corporation) and R version 4.4.2 (The R Foundation for Statistical Computing).

## Results

**FIGURE 1:**
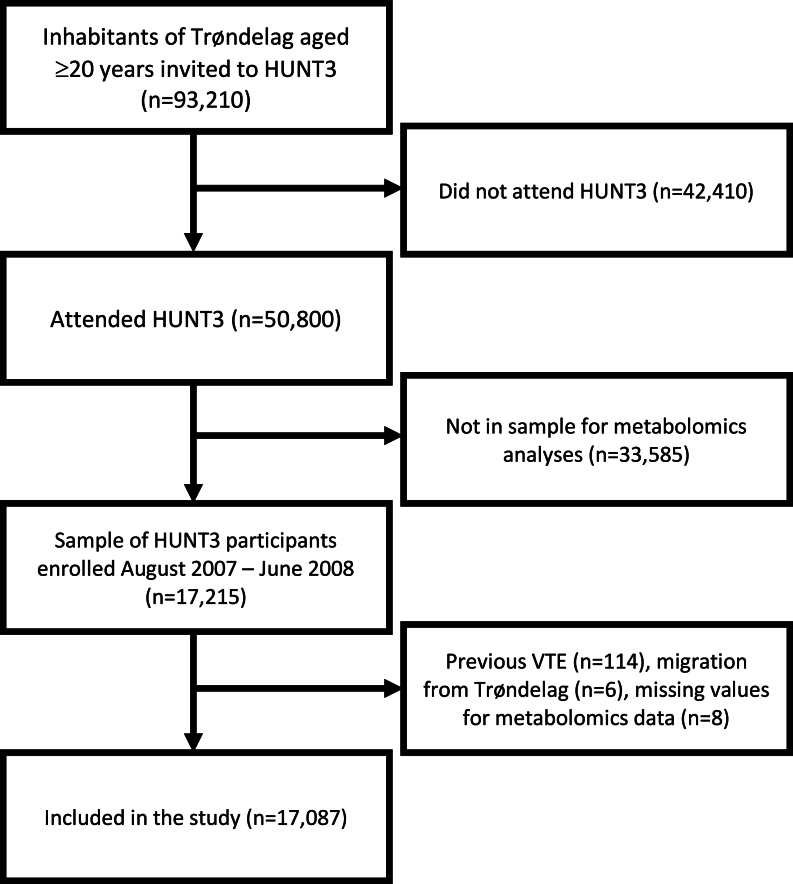
Flow chart describing the inclusion of participants in the present study. HUNT3, the third survey of the Trøndelag Health Study; VTE; venous thromboembolism.

**FIGURE 2:**
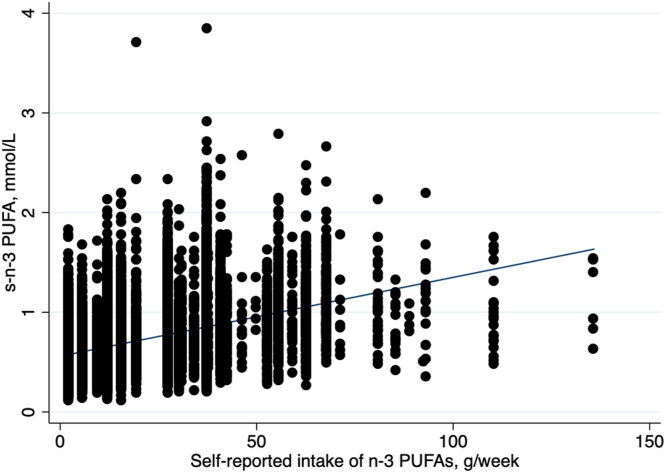
The relationship between self-reported intake of ω-3 polyunsaturated fatty acids (n–3 PUFAs) in grams per week and serum (s) n–3 PUFA concentrations in mmol/L. Scatter plot shown with a fitted linear regression line.

A flow chart visualizing the study population is shown in Figure 1. The mean age at HUNT3 participation was 52.3 ± 15.7 y, and 46.0% of the study population were men. The correlation coefficient for the correlation between estimated dietary intake of marine n–3 PUFAs and serum total n–3 PUFA concentrations was 0.45. The correlation coefficient for the correlation between estimated dietary intake of marine n–3 PUFAs and serum DHA concentrations was 0.51. Linear regression analysis showed that serum n–3 PUFA concentrations increased with increasing dietary intake of marine n–3 PUFAs (regression coefficient: 0.0079; 95% CI: 0.0076, 0.0082) with *R*^2^ = 0.19 (Figure 2). When estimated weekly intake of marine n–3 PUFAs based on self-reported questionnaires and n–3 PUFA concentrations was categorized into tertiles, κ statistic calculation showed that only 49.3% of participants were categorized in the same tertile for both measures with κ = 0.24.

The mean n–3 PUFA concentration was 0.73 ± 0.31 mmol/L. Baseline characteristics of the study population across tertiles of serum n–3 PUFA concentrations are shown in Table 1. Age, sex, BMI, history of cardiovascular disease or cancer, diabetes, smoking status, physical activity, and intake of fruits and vegetables varied across tertiles of n–3 PUFA concentrations.

**TABLE 1:** Baseline characteristics of the study population over tertiles of n–3 PUFA concentrations.

	Tertile of n–3 PUFA concentrations		
	T1 (*n* = 5696), <0.56 mmol/L	T2 (*n* = 5696), 0.56–0.81 mmol/L	T3 (*n* = 5695), ≥0.81 mmol/L
Age, y	42 (33.1, 53.6)	53.3 (42.8, 62.9)	60.4 (52.1, 68.6)
Male	2663 (46.8%)	2723 (47.8%)	2475 (43.5%)
Body mass index, kg/m^2^	25.6 (23.1, 28.6)	26.9 (24.4, 29.8)	27.3 (24.9, 30.0)
History of cancer	135 (2.4%)	286 (5.0%)	434 (7.6%)
History of cardiovascular disease	261 (4.6%)	449 (7.9%)	519 (9.1%)
Diabetes	147 (2.6%)	191 (3.4%)	253 (4.4%)
Daily or occasional smoker	1627 (29.2%)	1386 (25.0%)	1090 (19.7%)
Physical activity ≥1/wk	4201 (74.5%)	4335 (77.2%)	4464 (79.9%)
Self-reported intake of fruits/berries ≥1/d	2520 (44.3%)	2997 (52.7%)	3458 (60.7%)
Self-reported intake of vegetables ≥1/d	2182 (38.3%)	2826 (49.7%)	3258 (57.2%)
Self-reported n–3 PUFAs, g/wk	12.2 (2.3, 15.8)	15.8 (9.5, 37.4)	37.4 (15.8, 37.4)

Values are median (Q1, Q3) or *n* (%).

Abbreviations: n–3 PUFA, ω-3 polyunsaturated fatty acid; Q, quartile; T, tertile.

The study participants were followed for a total of 192,756 person-years, and 340 participants experienced a first-ever VTE event, resulting in an IR of 1.8 cases of VTE per 1000 person-years. The median follow-up time was 12.0 y (range: 0.04–13.2 y). The patient characteristics at VTE diagnosis are presented in Table 2. The median age at VTE diagnosis was 71.2 y, and 53% of the participants who experienced a VTE event were men. PE was the most common presentation (56% of VTE events). Among the VTE events, 65% were provoked, and cancer was a frequent provoking factor.

**TABLE 2:** Patient characteristics at first-ever VTE diagnosis.

	Total (*N* = 340)
Age at VTE, y	71.2 (61.7, 78.6)
Male	180 (52.9%)
Time from enrollment to VTE, y	6.9 (4.1, 9.6)
Deep vein thrombosis	150 (44.1%)
Pulmonary embolism	190 (55.9%)
Unprovoked VTE	118 (34.7%)
Provoked VTE	222 (65.3%)
VTE provoked by active cancer	89 (26.2%)

Values are median (Q1, Q3) or *n* (%).

Q, quartile; VTE, venous thromboembolism

The associations between n–3 PUFA concentrations and risk of VTE are presented in Table 3. There was an inverse association between serum n–3 PUFA concentrations and risk of future VTE. Subjects with serum n–3 PUFA concentrations in the highest tertile had an HR for overall VTE of 0.72 (95% CI: 0.54, 0.96) compared with those in the lowest tertile in the age-adjusted analysis. Further adjustment for sex, BMI, and history of cardiovascular disease or cancer had a negligible impact on risk estimate. The inverse association was particularly pronounced for provoked VTE and DVT, with 38% (HR: 0.62%; 95% CI: 0.44, 0.87%) and 39% (HR: 0.61%; 95% CI: 0.40, 0.93%) lower relative risk of VTE, respectively, when comparing the highest with the lowest tertile in the age-adjusted model. For unprovoked VTE and PE, a comparison of the highest and lowest tertiles yielded HRs of 1.00 (95% CI: 0.59, 1.67) for unprovoked VTE and 0.81 (95% CI: 0.55, 1.20) for PE in the age-adjusted model. The association between DHA concentrations and risk of overall VTE and VTE subgroups are presented in Supplemental Table 1 and showed similar results as those for total n–3 PUFAs.

**TABLE 3:** The association between total n–3 PUFA concentrations and risk of first-ever VTE (*N* = 17,087).

Concentration of n–3 PUFA, mmol/L	Person-years	VTE events	Crude incidence rate (95% CI), per 1000 person-years	HR model 1^1^ (95% CI)	HR model 2^2^ (95% CI)
Total VTE					
T1 <0.56	64,883	86	1.33 (1.07, 1.64)	1 (ref.)	1 (ref.)
T2 0.56, 0.81	64,382	118	1.83 (1.53, 2.20)	0.85 (0.64, 1.13)	0.83 (0.62, 1.10)
T3 ≥0.81	63,491	136	2.14 (1.81, 2.53)	0.72 (0.54, 0.96)	0.71 (0.54, 0.95)
*P* for trend^3^				0.02	0.02
Continuous^4^	192,756	340	1.76 (1.59, 1.96)	0.88 (0.79, 0.99)	0.88 (0.78, 0.99)
Unprovoked VTE					
T1 <0.56	64,883	24	0.37 (0.25, 0.55)	1 (ref.)	1 (ref.)
T2 0.56, 0.81	64,382	42	0.65 (0.48, 0.88)	1.11 (0.66, 1.87)	1.06 (0.63, 1.78)
T3 ≥0.81	63,491	52	0.82 (0.62, 1.07)	1.00 (0.59, 1.67)	0.99 (0.59, 1.66)
*P* for trend^3^				0.88	0.91
Continuous^4^	192,756	118	0.61 (0.51, 0.73)	1.03 (0.86, 1.23)	1.03 (0.86, 1.24)
Provoked VTE					
T1 <0.56	64,883	62	0.96 (0.75, 1.23)	1 (ref.)	1 (ref.)
T2 0.56, 0.81	64,382	76	1.18 (0.94, 1.48)	0.76 (0.54, 1.06)	0.74 (0.53, 1.05)
T3 ≥0.81	63,491	84	1.32 (1.07, 1.64)	0.62 (0.44, 0.87)	0.61 (0.43, 0.87)
*P* for trend^3^				0.01	0.01
Continuous^4^	192,756	222	1.15 (1.01, 1.31)	0.80 (0.69, 0.93)	0.80 (0.69, 0.93)
PE					
T1 <0.56	64,883	44	0.68 (0.50, 0.91)	1 (ref.)	1 (ref.)
T2 0.56, 0.81	64,382	62	0.96 (0.75, 1.24)	0.84 (0.57, 1.25)	0.81 (0.55, 1.21)
T3 ≥0.81	63,491	84	1.32 (1.07, 1.64)	0.81 (0.55, 1.20)	0.82 (0.55, 1.20)
*P* for trend^3^				0.34	0.37
Continuous^4^	192,756	190	0.99 (0.86, 1.14)	0.92 (0.80, 1.07)	0.93 (0.80, 1.08)
DVT					
T1 <0.56	64,883	42	0.65 (0.48, 0.88)	1 (ref.)	1 (ref.)
T2 0.56, 0.81	64,382	56	0.87 (0.67, 1.13)	0.87 (0.58, 1.31)	0.84 (0.56, 1.27)
T3 ≥0.81	63,491	52	0.82 (0.62, 1.07)	0.61 (0.40, 0.93)	0.59 (0.39, 0.92)
*P* for trend^3^				0.02	0.02
Continuous^4^	192,756	150	0.78 (0.66, 0.91)	0.83 (0.69, 0.99)	0.82 (0.68, 0.98)

Cox proportional hazards regression models with age as time scale were used to estimate HRs and 95% CIs for the associations between total serum n–3 PUFA concentrations and risk of first-ever VTE. HRs were estimated for total first-ever VTE, for PE and DVT separately, and for provoked and unprovoked VTE separately.

Abbreviations: CI, confidence interval; DVT, deep vein thrombosis; HR, hazard ratio; n–3 PUFA, ω-3 polyunsaturated fatty acid; PE, pulmonary embolism; ref., reference; T, tertile; VTE, venous thromboembolism.

1 Adjusted for age (as time scale).

2 Adjusted for age (as time scale), sex, body mass index, and history of cardiovascular disease or cancer.

3 In the *P* for trend analysis, n–3 PUFA in tertiles, with the values 1, 2, and 3, was entered as a discrete variable in the Cox proportional hazards regression analysis.

4 n–3 PUFA was entered as a continuous variable in units of standard deviations of 0.31 mmol/L in the Cox proportional hazards regression analysis.

**FIGURE 3:**
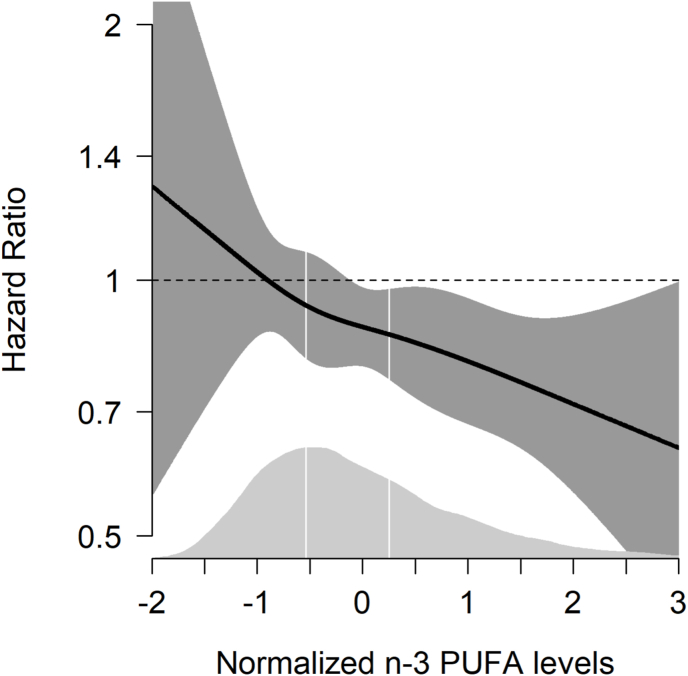
Hazard ratios (HRs) for overall venous thromboembolism as a function of normalized serum ω-3 polyunsaturated fatty acid (n–3 PUFA) concentrations in a generalized additive regression model. Total n–3 PUFAs were modeled with a smoothing spline fit in a Cox proportional hazards regression model. HRs were adjusted for age, sex, body mass index, cancer, and arterial cardiovascular disease at baseline. n–3 PUFA concentrations were standardized to a mean value of 0 and a standard deviation of 1 before entering the analyses. The solid lines depict HRs surrounded by shaded areas (gray) representing 95% confidence intervals. The distributions of serum-normalized total n–3 PUFA concentrations are shown as density plots (light gray) at the bottom, and white vertical lines indicate tertile cutoffs.

The HRs for overall VTE by serum n–3 PUFAs, entered as a spline, are shown in Figure 3. There was an inverse, almost linear, relationship between serum n–3 PUFA concentrations and overall VTE risk. Notably, the HR estimates at more extreme serum n–3 PUFA concentrations had wide 95% CIs due to limited number of individuals with extreme values. To consider the possibility of underestimating the true association due to regression dilution bias, we estimated HRs of overall VTE (highest compared with lowest tertile of n–3 PUFA) as a function of follow-up time (Supplemental Figure 1). The HRs for overall VTE by high serum n–3 PUFA concentrations remained rather stable throughout the entire follow-up time.

In a sensitivity analysis, we investigated the association between serum n–3 PUFA concentrations, DHA concentrations, and risk of VTE that was not provoked by active cancer. The results were similar to those found in the main analyses (data not shown).

We found no association between estimated weekly intake of marine n–3 PUFAs based on self-reported questionnaires and risk of overall VTE (Supplemental Table 2). However, there was an association between higher self-reported intake of marine n–3 PUFAs and higher risk of unprovoked VTE.

## Discussion

In this population-based cohort, higher serum n–3 PUFA concentrations were associated with decreased risk of future incident overall VTE. The risk estimates remained unchanged after multivariable adjustments. The inverse relationship between serum n–3 PUFA concentrations and risk of overall VTE was almost linear when serum n–3 PUFA concentration was entered as a spline. The inverse relationship was most pronounced for provoked VTE and DVT. Our findings suggest that high serum n–3 PUFA concentrations are protective against risk of future VTE.

Results from 2 retrospective case-control studies reported lowered EPA concentrations in individuals with VTE [28,29], and a study derived from the UK Biobank, including both incident and prevalent VTE, showed an inverse association between serum DHA, total n–3 PUFA concentrations, and VTE [30]. Even though these study designs do not allow for causal inference due to the possibility of reverse causation (e.g., dietary changes because of the VTE event), the results imply an inverse association between serum n–3 PUFA concentrations and VTE risk. Although the cohort design used in the present study facilitated a proper time sequence between n–3 PUFA concentration and VTE, the apparent protective effect of n–3 PUFAs on VTE risk, even appearing after major putative confounders were accounted for, might be explained by residual confounding (i.e., unmeasured and unrecognized confounders) due to the observational nature of our study. Presence of residual confounding in our study is supported by the results of a 2-sample Mendelian randomization study [30] of data from 114,999 individuals regarding genome-wide association data as well as serum concentrations of DHA and total n–3 PUFAs concentrations. They identified 46 single nucleotide polymorphisms that explained 6.5% of serum DHA concentrations, and the odds ratio for 1-unit increase in genetically predicted DHA for prevalent and incident VTE was 1.07 (95% CI: 0.96, 1.19) using data from multiple cohorts, including 1,153,768 individuals of European ancestry. Similar results were found for genetically predicted total n–3 PUFA concentrations [30]. As n–3 PUFAs are essential fatty acids acquired by intake of marine food and supplements, the limited ability of gene variants to capture dietary habits might partly explain the apparent discrepancy between the results of the Mendelian randomization study and our present findings.

The inverse association between serum n–3 PUFA concentrations and VTE risk observed in the present study was mainly driven by risk of provoked VTE and DVT. Accordingly, a previous cohort study found an association between higher self-reported intake of marine n–3 PUFAs and lower risk of provoked VTE and provoked PE in particular [34]. The explanations for these observations are unknown but imply that high n–3 PUFA concentrations reduce risk of a VTE event in the presence of provoking factors (e.g., immobilization). Alternatively, provoking factors might occur less frequently in individuals with higher n–3 PUFA concentrations, at least in part due to a healthier lifestyle.

We found that the association between n–3 PUFA concentrations and VTE risk seemed to be more pronounced for DVT than for PE. Studies investigating the association between n–3 PUFAs and DVT specifically are scarce. However, a previous Norwegian study exploring the association between estimated intake of n–3 PUFAs on risk of future VTE reported an inverse association between n–3 PUFA intake and PE, but not DVT [34].

In our study, high estimated weekly intake of marine n–3 PUFAs based on self-reported fish consumption was not associated with overall VTE risk and was associated with an elevated risk of unprovoked VTE. In the food frequency questionnaire used in our study, high-fat fish on bread or for dinner (salmon, trout, herring, mackerel, haddock) along with dietary supplements such as cod liver oil and ω-3 capsules were registered. Data from the frequency questionnaire of fatty fish intake and n–3 PUFA-enriched supplements were used along with recommendations from the Norwegian Directorate of Health regarding standard serving size, and n–3 PUFA content was used to estimate the weekly intake of marine n–3 PUFAs. Although serum total n–3 PUFA concentrations is an accurate measure reflecting the dietary intake of marine-derived food and supplements, estimated weekly intake of marine n–3 PUFAs is dependent on the ability of the study participants to accurately report their dietary habits and the ability of the self-reported questionnaires to capture the separate sources of marine food and supplements, along with serving sizes and content of n–3 PUFAs. The content of n–3 PUFAs is much higher in fatty than lean fish, varies between different species of fatty fish and between wild caught fish and farmed fish, as well as between different n–3 PUFA-enriched supplements [25,26]. Both the estimated correlation coefficient and κ score for the relationship between dietary intake of marine n–3 PUFAs and serum n–3 PUFA concentrations show that there is far from perfect agreement between the 2 measures. Thus, it is likely that the lack of association between dietary intake of n–3 PUFAs and VTE risk could be explained by nondifferential misclassifications of n–3 PUFA intake, with subsequent underestimation of the true association.

Our findings of an inverse relationship between serum n–3 PUFAs concentrations and risk of future VTE, along with similar findings in 2 case-control studies [28,29] and 1 large population-based study [30], indicate a protective role of dietary intake of n–3 PUFAs on VTE risk. This notion is supported by accumulating evidence for antithrombotic properties of n–3 PUFAs, such as mitigation of thrombin generation [11], platelet function [12,13], platelet-vessel wall interaction [40], and endogenous coagulation activation, assessed by plasma D-dimer concentrations [41], all of which are implicated in the pathogenesis of VTE. Although the costs incurred might be prohibitive, randomized controlled studies assessing dietary interventions with n–3 PUFAs on incident or recurrent VTE are needed to prove causality. Our results contribute to the growing evidence of a protective effect of dietary intake of n–3 PUFAs on VTE risk and should be included in the evidence base for future nutritional guidelines and advice.

A strength of the present study is the large, population-based cohort in which all first-ever VTE events were validated, and only objectively verified events were included as outcomes. We included both men and women over a wide age range. The population-based study design and the wide inclusion criteria ensures high generalizability and external validity. Serum concentrations of relevant fatty acids were objectively measured in samples collected at baseline. A washout study after n–3 PUFA supplementation indicated that a single measurement of serum n–3 PUFA concentration presumably reflects the n–3 PUFA intake during the past months [42]. Nonetheless, the long follow-up time could be considered a limitation as changes in dietary habits over time could introduce regression dilution bias and lead to underestimations of the true associations between n–3 PUFAs and risk of first-ever VTE. However, the regression dilution plot showed that the HR was relatively constant over time. Another limitation of the present study is that the samples were not flushed with nitrogen after collection to avoid oxidation. Lipid oxidation could lead to an underestimation of true n–3 PUFA concentrations and a risk of biasing our results toward the null. Furthermore, it would have been preferable to present the associations between individual n–3 PUFAs and risk of VTE, in addition to the presented data on total n–3 PUFA concentrations and DHA concentrations. Finally, although we adjusted for potential confounders, the observational design of the study implies that residual confounding cannot be ruled out, and conclusions about causality cannot be drawn.

In conclusion, we found an association between higher serum concentrations of n–3 PUFAs and lower risk of future incident overall VTE and provoked VTE and DVT in particular. Our findings support that high intake of n–3 PUFAs may protect against future risk of VTE.

## Author contributions

The authors’ responsibilities were as follows – JBH: designed the research; JBH, BOÅ, GFG, KHW, NT, GDS, KH, BB: conducted the research and provided essential materials; MJ, KDH: analyzed the data; MJ, JBH, SKB, KDH: interpreted the results; MJ: drafted the manuscript; JBH, SKB, GFG, KHW, NT, GDS, KDH, KH, BB, BOÅ: revised the manuscript for critical content; and all authors: read and approved the final manuscript.

## Data availability

Data described in the manuscript will not be made available due to restrictions in the Ethics approval. Access to data from the HUNT study can be obtained by application to the HUNT administration (https://www.ntnu.edu/hunt/data).

## Funding

The authors reported no funding received for this study.

## Conflict of interest

The authors report no conflicts of interest.
