# Supplementary material for: High concentrations of polyunsaturated n–3 fatty acids in serum are inversely associated with risk of future incident venous thromboembolism – the HUNT cohort study
Source: Am J Clin Nutr. 2025 Aug 18;122(5):1195–203. doi: 10.1016/j.ajcnut.2025.08.008 (PMC12799366; doi:10.1016/j.ajcnut.2025.08.008)
Supplement: Multimedia component 1 [file mmc1.pdf]

Johansson, M., High concentrations of polyunsaturated n-3 fatty acids in serum are inversely associated with risk of future incident venous thromboembolism – The HUNT cohort study

Supplementary Figure 1. Plot of estimated hazard ratios (HRs) for overall venous thromboembolism (VTE) as a function of maximum time from blood sampling in the third HUNT survey to the VTE event.

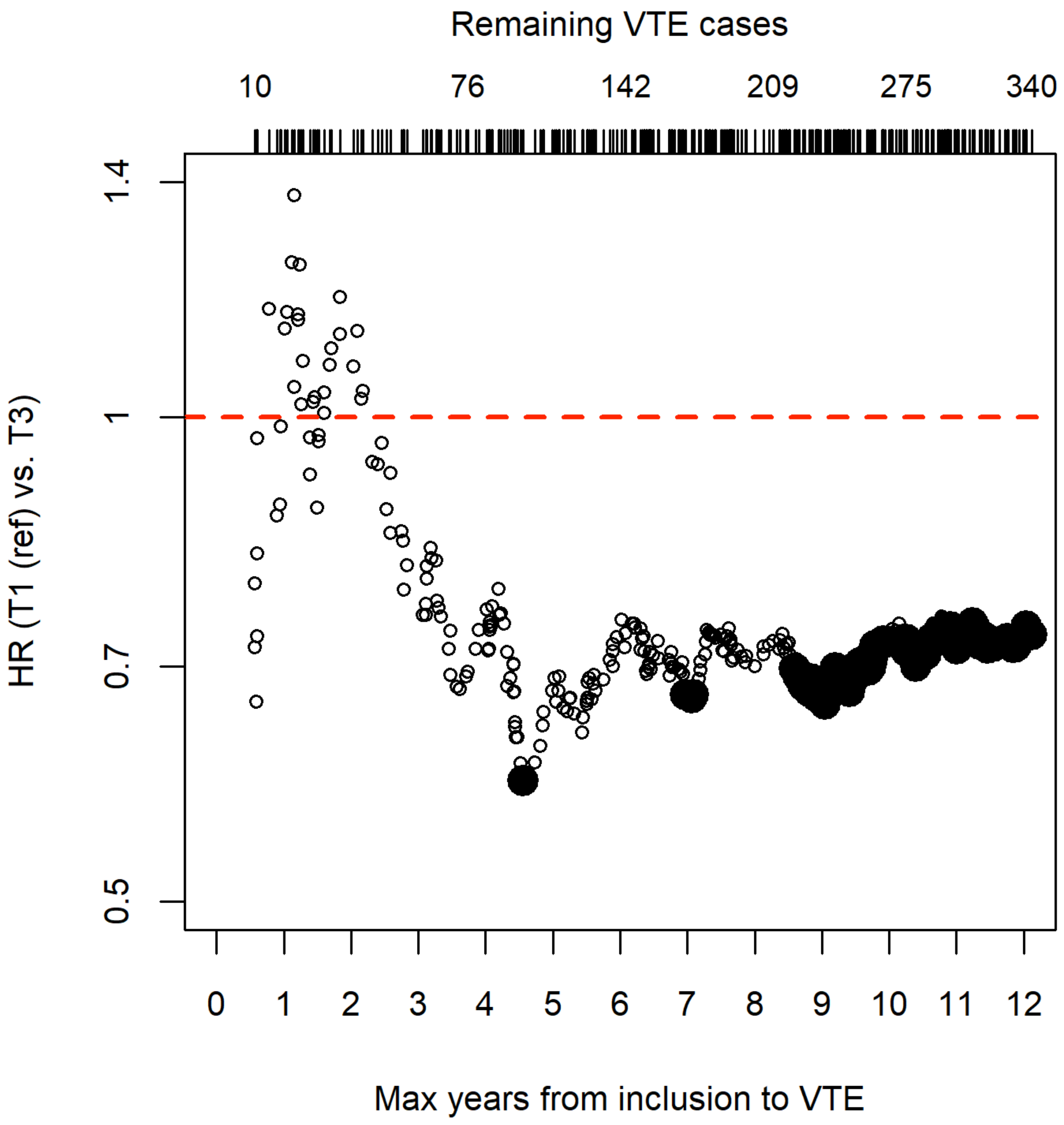

All analyses were adjusted for age (as time scale), sex, body mass index and history of cardiovascular disease and cancer at baseline. Subjects with serum n-3 PUFA levels in the

highest tertile (T3) were compared with those with n-3 PUFA levels in the lowest tertile (T1, reference category). Large, solid circles indicate HRs with P-value<0.05. The number of VTE events are depicted above the plot. Maximum follow-up time from blood sampling to the VTE events was restricted while keeping all cohort members in the analyses. The Cox regression proportional hazards regression analyses on time restrictions were set to require at least 10 VTE events, and HRs were generated at every time point a new VTE event occurred and plotted as a function of this maximum time.

HR, hazard ratio; HUNT, Trøndelag Health Study; n-3 PUFA, omega-3 polyunsaturated fatty acid; T, tertile; VTE, venous thromboembolism

Supplementary Table 1. The association between docosahexaenoic acid concentrations and risk of first-ever VTE (n=17,087)

Concentration of docosahexaenoic acid, mmol/L	Person-years	VTE events	Crude incidence rate (95% CI), per 1000 person-years	Hazard ratio model 1[1] (95% CI)	Hazard ratio model 2[2] (95% CI)
Total VTE					
T1 < 0.26	64,821	90	1.39 (1.13, 1.71)	1 (ref.)	1 (ref.)
T2 0.26–0.35	64,480	112	1.74 (1.44, 2.09)	0.82 (0.62, 1.09)	0.85 (0.63, 1.12)
T3 ≥ 0.35	63,455	138	2.17 (1.84, 2.57)	0.71 (0.54, 0.94)	0.77 (0.58, 1.02)
P for trend[3]				0.02	0.07
Continuous[4]	192,755	340	1.76 (1.59, 1.96)	0.87 (0.78, 0.97)	0.90 (0.80, 1.01)
Unprovoked VTE					
T1 < 0.26	64,821	26	0.40 (0.27, 0.59)	1 (ref.)	1 (ref.)
T2 0.26–0.35	64,480	32	0.50 (0.35, 0.70)	0.83 (0.49, 1.42)	0.87 (0.51, 1.49)
T3 ≥ 0.35	63,455	60	0.95 (0.73, 1.22)	1.09 (0.66, 1.78)	1.24 (0.75, 2.05)
P for trend[3]				0.55	0.26
Continuous[4]	192,755	118	0.61 (0.51, 0.73)	1.02 (0.86, 1.21)	1.07 (0.90, 1.27)
Provoked VTE					
T1 < 0.26	64,821	64	0.99 (0.77, 1.26)	1 (ref.)	1 (ref.)
T2 0.26–0.35	64,480	80	1.24 (1.00, 1.54)	0.81 (0.58, 1.14)	0.83 (0.59,1.16)
T3 ≥ 0.35	63,455	78	1.23 (0.98, 1.53)	0.56 (0.40, 0.80)	0.59 (0.42, 0.84)
P for trend[3]				0.001	0.003
Continuous[4]	192,755	222	1.15 (1.01, 1.31)	0.79 (0.68, 0.91)	0.81 (0.70, 0.93)
PE					
T1 < 0.26	64,821	45	0.69 (0.52, 0.93)	1 (ref.)	1 (ref.)
T2 0.26–0.35	64,480	62	0.96 (0.75, 1.23)	0.87 (0.59, 1.29)	0.89 (0.60, 1.33)
T3 ≥ 0.35	63,455	83	1.31 (1.05, 1.62)	0.80 (0.55, 1.17)	0.87 (0.59, 1.29)
P for trend[3]				0.26	0.52
Continuous[4]	192,755	190	0.99 (0.86, 1.14)	0.90 (0.78, 1.04)	0.93 (0.80, 1.08)
DVT					
T1 < 0.26	64,821	45	0.69 (0.52, 0.93)	1 (ref.)	1 (ref.)
T2 0.26–0.35	64,480	50	0.78 (0.59, 1.02)	0.76 (0.51, 1.15)	0.79 (0.52, 1.19)
T3 ≥ 0.35	63,455	55	0.87 (0.67, 1.13)	0.61 (0.40, 0.93)	0.65 (0.43, 0.99)
P for trend[3]				0.02	0.05
Continuous[4]	192,755	150	0.78 (0.66, 0.91)	0.83 (0.70, 0.99)	0.86 (0.72, 1.02)

CI, confidence interval; DVT, deep vein thrombosis; HR; hazard ratio; PE, pulmonary embolism; T, tertile; VTE, venous thromboembolism

Cox proportional hazards regression models with age as time scale were used to estimate hazard ratios (HRs) and 95% CIs for the associations between total serum docosahexaenoic acid concentrations and risk of first-ever VTE. HRs were estimated for total first-ever VTE, for PE and DVT separately, and for provoked and unprovoked VTE separately.

[1]Adjusted for age (as time scale)

[2]Adjusted for age (as time scale), sex, body mass index, history of cardiovascular disease and cancer

[3]In the *P* for trend analysis, docosahexaenoic acid in tertiles, with the levels 1, 2 and 3, was entered as a discrete variable in the Cox proportional hazards regression analysis.

[4]Docosahexaenoic acid was entered as a continuous variable in units of standard deviations of 0.12 mmol/L in the Cox proportional hazards regression analysis.

Supplementary Table 2. The association between self-reported intake of marine n-3 PUFAs and risk of first-ever VTE

Intake of n-3 PUFAs, g/week	Person-years	VTE events	Crude incidence rate (95% CI), per 1000 person-years	Hazard ratio model 1[1] (95% CI)	P for trend	Hazard ratio model 2[2] (95% CI)	P for trend
Total VTE							
T1 < 15.8	88,315	118	1.34 (1.12, 1.60)	1 (ref.)		1 (ref.)	
T2 15.8–30.5	39,887	59	1.48 (1.15, 1.91)	0.89 (0.65, 1.22)	0.93	0.93 (0.68, 1.28)	0.64
T3 ≥ 30.5	60,297	153	2.54 (2.17, 2.97)	0.98 (0.76, 1.27)		1.06 (0.82, 1.37)	
Continuous[3]	188,499	330	1.75 (1.57, 1.95)	0.96 (0.86, 1.08)		1.00 (0.89, 1.11)	
Unprovoked VTE							
T1 < 15.8	88,315	30	0.34 (0.24, 0.49)	1 (ref.)		1 (ref.)	
T2 15.8–30.5	39,887	19	0.48 (0.30, 0.75)	1.16 (0.65, 2.07)	0.04	1.27 (0.71, 2.28)	0.01
T3 ≥ 30.5	60,297	62	1.03 (0.80, 1.32)	1.61 (1.01, 2.56)		1.87 (1.16, 3.01)	
Continuous[3]	188,499	111	0.59 (0.49, 0.71)	1.14 (0.96, 1.36)		1.20 (1.01, 1.44)	
Provoked VTE							
T1 < 15.8	88,315	88	1.00 (0.81, 1.23)	1 (ref.)		1 (ref.)	
T2 15.8–30.5	39,887	40	1.00 (0.74, 1.37)	0.80 (0.55, 1.17)	0.12	0.83 (0.57, 1.20)	0.20
T3 ≥ 30.5	60,297	91	1.51 (1.23, 1.85)	0.78 (0.57, 1.06)		0.81 (0.60, 1.11)	
Continuous[3]	188,499	219	1.16 (1.02, 1.33)	0.88 (0.76, 1.01)		0.90 (0.78, 1.03)	
PE							
T1 < 15.8	88,315	70	0.79 (0.63, 1.00)	1 (ref.)		1 (ref.)	
T2 15.8–30.5	39,887	29	0.73 (0.51, 1.05)	0.72 (0.47, 1.11)	0.39	0.75 (0.49, 1.17)	0.68
T3 ≥ 30.5	60,297	85	1.41 (1.14, 1.74)	0.86 (0.61, 1.20)		0.92 (0.66, 1.30)	
Continuous[3]	188,499	184	0.98 (0.84, 1.13)	0.93 (0.80, 1.08)		0.96 (0.82, 1.11)	
DVT							
T1 < 15.8	88,315	48	0.54 (0.41, 0.72)	1 (ref.)		1 (ref.)	
T2 15.8–30.5	39,887	30	0.75 (0.53, 1.08)	1.15 (0.73, 1.83)	0.41	1.20 (0.76, 1.91)	0.24
T3 ≥ 30.5	60,297	68	1.13 (0.89, 1.43)	1.18 (0.80, 1.75)		1.27 (0.86, 1.89)	
Continuous[3]	188,499	146	0.77 (0.66, 0.91)	1.02 (0.86, 1.20)		1.05 (0.89, 1.24)	

CI, confidence interval; DVT, deep vein thrombosis; HR, hazard ratio; n-3 PUFA, omega-3 polyunsaturated fatty acid; PE, pulmonary embolism; T, tertile; VTE, venous thromboembolism.

Cox proportional hazards regression models with age as time scale were used to estimate hazard ratios (HRs) and 95% CIs for the associations between self-reported weekly intake of n-3 PUFAs and risk of first-ever VTE. HRs were estimated for total first-ever VTE, for PE and DVT separately, and for provoked and unprovoked VTE separately.

[1]Adjusted for age (as time scale)

[2]Adjusted for age (as time scale), sex, body mass index, history of cardiovascular disease and cancer

[3]n-3 PUFA was entered as a continuous variable in units of standard deviations of 17.13 g/week in the Cox proportional hazards regression analysis
